# Neutralizing Staphylococcus aureus Virulence with AZD6389, a Three mAb Combination, Accelerates Closure of a Diabetic Polymicrobial Wound

**DOI:** 10.1128/msphere.00130-22

**Published:** 2022-06-01

**Authors:** Christine Tkaczyk, Omari Jones-Nelson, Yue Yue Shi, David E. Tabor, Lily Cheng, Tianhui Zhang, Bret R. Sellman

**Affiliations:** a Early Vaccines and Immune Therapies, BioPharmaceuticals R&D, AstraZeneca, Gaithersburg, Maryland, USA; b Oncology Safety, Clinical Pharmacology and Safety Sciences, R&D, AstraZeneca, Gaithersburg, Maryland, USA; c Data Sciences and Quantitative Biology AstraZeneca, Gaithersburg, Maryland, USA; University of Nebraska Medical Center

**Keywords:** Staphylococcus aureus, alpha toxin, clumping factor A, diabetic foot, leukocidins, monoclonal antibodies, polymicrobial infections, wound healing

## Abstract

Nonhealing diabetic foot ulcers (DFU), a major complication of diabetes, are associated with high morbidity and mortality despite current standard of care. Since Staphylococcus aureus is the most common pathogen isolated from nonhealing and infected DFU, we hypothesized that S. aureus virulence factors would damage tissue, promote immune evasion and alter the microbiome, leading to bacterial persistence and delayed wound healing. In a diabetic mouse polymicrobial wound model with S. aureus, Pseudomonas aeruginosa, and Streptococcus pyogenes, we report a rapid bacterial proliferation, prolonged pro-inflammatory response and large necrotic lesions unclosed for up to 40 days. Treatment with AZD6389, a three-monoclonal antibody combination targeting S. aureus alpha toxin, 4 secreted leukotoxins, and fibrinogen binding cell-surface adhesin clumping factor A resulted in full skin re-epithelization 21 days after inoculation. By neutralizing multiple virulence factors, AZD6389 effectively blocked bacterial agglutination and S. aureus-mediated cell killing, abrogated S. aureus-mediated immune evasion and targeted the bacteria for opsonophagocytic killing. Neutralizing S. aureus virulence not only facilitated S. aureus clearance in lesions, but also reduced S. pyogenes and P. aeruginosa numbers, damaging inflammatory mediators and markers for neutrophil extracellular trap formation 14 days post initiation. Collectively, our data suggest that AZD6389 holds promise as an immunotherapeutic approach against DFU complications.

**IMPORTANCE** Diabetic foot ulcers (DFU) represent a major complication of diabetes and are associated with poor quality of life and increased morbidity and mortality despite standard of care. They have a complex pathogenesis starting with superficial skin lesions, which often progress to deeper tissue structures up to the bone and ultimately require limb amputation. The skin microbiome of diabetic patients has emerged as having an impact on DFU occurrence and chronicity. DFU are mostly polymicrobial, and the Gram-positive bacterium Staphylococcus aureus detected in more than 95% of cases. S. aureus possess a collection of virulence factors which participate in disease progression and may facilitate growth of other pathogens. Here we show in a diabetic mouse wound model that targeting some specific S. aureus virulence factors with a multimechanistic antibody combination accelerated wound closure and promoted full skin re-epithelization. This work opens promising new avenues for the treatment of DFU.

## INTRODUCTION

The incidence of diabetes has increased dramatically over the past two decades, affecting more than 10% of the population worldwide (1). Diabetes is associated with a high morbidity and mortality, and diabetic foot ulcers (DFU) are a major complication of the disease with ~$13 billion annual cost to the health care system in the United States alone (2). Defects in lower extremity blood flow, neuropathy, poor glycemic control and an abnormal host immune response are associated with delayed healing, chronic wounds and recurrence (3, 4). DFU pathophysiology is complex (5) and current standard of care (SOC) relies on wound debridement, management of infection, revascularization procedures when required and off-loading of the ulcer along with appropriate glycemic control (6, 7). However, recurrence rates and severe complications indicate these approaches are not sufficient to achieve complete wound re-epithelization and prevent recurrence (5). To address this unmet medical need, several experimental molecules or approaches are currently under clinical evaluation to improve upon SOC. This includes topical administration of growth factors, *in situ* gene therapy with vascular endothelial growth factor (VEGF) or hepatocyte growth factor (HGF) (reviewed in Ref. 8), a wound dressing containing metalloproteinase inhibitors (9) or topical oxygen therapy (10). To date, only topical platelet derived growth factor (PDGF) has been approved by the FDA but may only be used for neuropathic ulcers with adequate peripheral circulation (11).

The skin microbiome has emerged as playing an integral role in DFU occurrence and healing (12–14). All DFU are colonized with microorganisms, but only ~50% are considered infected based on presence of clinical signs such as redness, purulence, swelling, warmth, pain, or induration (5, 15). Microbiological analysis of these foot ulcers by 16S rRNA sequencing demonstrated that DFU are polymicrobial, with Staphylococcus aureus being the most commonly identified pathogen in western countries and its presence was linked to nonhealing ulcers (6, 15–17). Other bacteria, such as P. aeruginosa and S. pyogenes are frequently detected along with S. aureus (18–21).

S. aureus is a Gram-positive opportunistic pathogen that causes a variety of infections, including surgical site infections, skin and soft tissue infections, bacteremia, and pneumonia (22) and can exhibit resistance to available antibiotic therapy. Consequently, alternative approaches to antibacterial therapy are being explored such as immunotherapy with specific monoclonal antibodies (MAbs) (23–25). S. aureus differentially expresses a variety of virulence factors in response to its environment and stage of infection to modify and evade a protective host immune response (26, 27). Among those, S. aureus secretes several pore-forming toxins including alpha toxin (AT) and the bicomponent leukotoxins (Leuk) which together lyse immune cells, induce a strong pro-inflammatory response and promote bacterial dissemination by damaging tissue and increasing vascular permeability (28, 29). Additionally, the S. aureus genome harbors a collection of microbial surface components recognizing adhesive matrix molecules (MSCRAMMs) which facilitate bacterial attachment to exposed extracellular matrix molecules leading to bacterial agglutination, biofilm formation and immune evasion (30). Therefore, we hypothesized that some S. aureus virulence factors would promote DFU colonization and create a local environment permissive for pathogen outgrowth resulting in chronic, nonhealing wounds. To determine if targeting S. aureus was beneficial in the healing process and could accelerate wound closure, we developed a polymicrobial wound model in diabetic mice with S. aureus and two other pathogens, P. aeruginosa and S. pyogenes, and tested the efficacy of anti-S. aureus AZD6389 MAb combination. These last two organisms were selected based on their prevalence in polymicrobial DFU along with S. aureus, and as primary causes of infection and delayed wound healing (31–34). AZD6389, a multimechanistic combination of three MAbs, targets S. aureus virulence factors AT, 4 bicomponent leukotoxins (LukSF/LukED/HlgAB/HlgCB) and the fibrinogen binding surface expressed MSCRAMM, clumping factor A (ClfA). These MAbs were identified for their ability to respectively inhibit *in vitro* AT-hemolytic activity (35), leukotoxins cytolytic activity, ClfA-mediated blood agglutination and bacteria opsonophagocytic killing (25). Together, this combination provides broad strain coverage in multiple S. aureus disease models (25, 36, 37). We found that diabetic animals challenged with a mixture of S. aureus/S. pyogenes/P. aeruginosa developed large, unhealed lesions resulting in part from bacterial proliferation, sustained local inflammation and neutrophil extracellular trap (NET) formation. AZD6389 administration accelerated bacterial clearance, decreased inflammation and reduced markers of NET formation leading to more rapid wound closure. Together, our results demonstrated that S. aureus virulence factors act in concert to create an environment permissive to pathogen outgrowth and promote the persistence of a chronic wound. Targeting S. aureus with the AZD6389 may open new avenues for the treatment of DFU.

## RESULTS

### Diabetic mice fail to heal wounds after polymicrobial inoculation.

**FIG 1:**
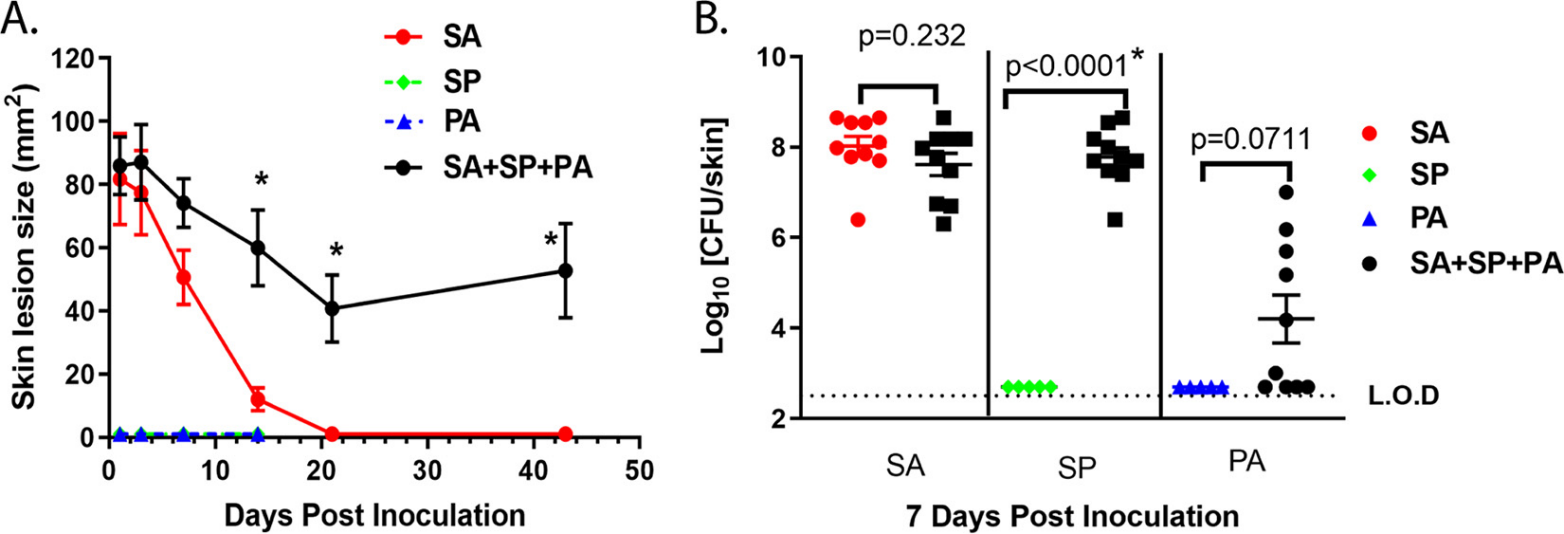
S. aureus (SA), P. aeruginosa (PA) and S. pyogenes (SP) mixed infection in the diabetic polymicrobial wound. (A) Skin lesion sizes induced by ID injection of SA SF8300 (1.0 × 10^7^ CFU), SP (10 CFU), PA (1.0 × 10^5^ CFU) or SA/SP/PA (*n* = 10 per group). Lesions were measured at indicated times and graphed as mean values ± standard error of the mean (SEM). Statistical significance for lesion sizes between two groups was determined using a Vardi’s AUC test (two sample tests for growth under the curve dependent right censoring) and considered statistically different if *P < *0.05. PA vs SA *P = *0.012; PA vs SA/PA/SP *P = *0.0036; SA vs SA+SP+PA *P = *0.005; SA vs SP *P = *0.0012; SP vs SA+PA+SP *P = *0.0036 (B) S. aureus, S. pyogenes and P. aeruginosa recovered from skin lesions of mice injected as described above after 7 days. Significant difference for CFU was analyzed with a Mann-Whitney test, and considered statistically different if *P < *0.05 as indicated with a (*).

**FIG 2:**
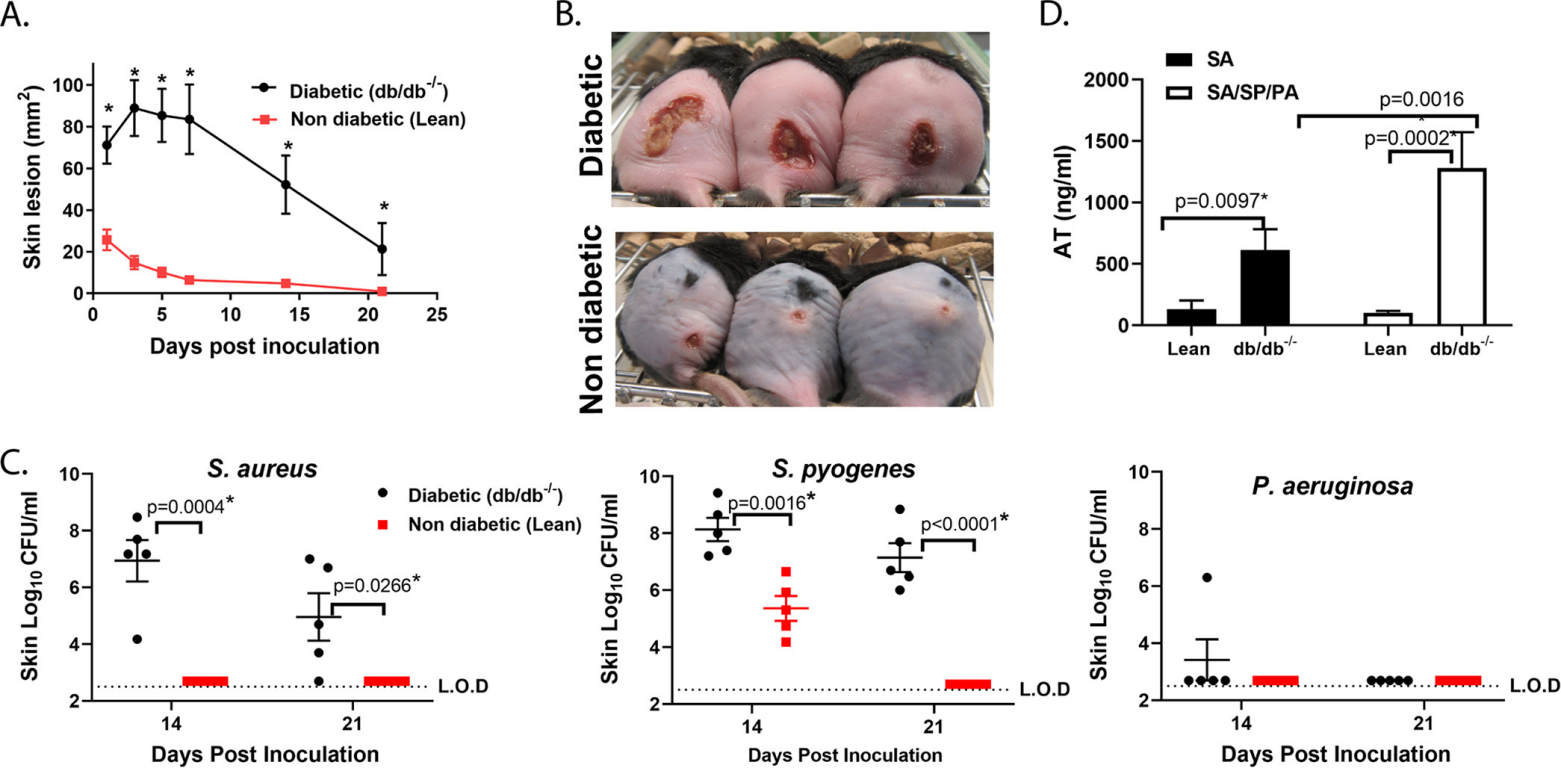
Polymicrobial wound healing and bacterial clearance is delayed in diabetic mice. (A) Skin lesion sizes of diabetic and nondiabetic mice (*n* = 10 per group) i.d. injected with S. aureus SF8300 (SA) - (1.0 × 10^7^ CFU)/P. aeruginosa (PA) (1.0 × 10^5^ CFU)/S. pyogenes (SP) (10 CFU). Lesions were measured at indicated times and graphed as mean values ± SEM. Statistical significance for lesion sizes between diabetic and nondiabetic mice injected with S. aureus/S. pyogenes/P. aeruginosa was determined using a Vardi’s AUC test (two sample tests for growth under the curve dependent right censoring) and considered statistically different if *P < *0.05 as indicated with a (*). (B) Pictures of representative skin lesions 14 days after i.d. inoculation. (C) S. aureus, S. pyogenes and P. aeruginosa recovered from skin lesions after 14 or 21 days from mice inoculated as indicated in (A). Data are represented at mean values ± SEM. Statistical difference for CFU was analyzed with a Mann-Whitney test, and considered statistically different if *P < *0.05 as indicated with a (*). (D) Skins was harvested 24 h postchallenge with S. aureus or S. aureus/S. pyogenes/P. aeruginosa, homogenized and analyzed by ELISA for AT content (ng/mL). Statistical difference between group was analyzed with a Mann-Whitney *t* test, and considered statistically different if *P < *0.05.

S. aureus is highly prevalent in DFU and is frequently present with other pathogens including S. pyogenes and P. aeruginosa (12, 38, 39). To better understand polymicrobial DFU pathogenesis and to determine if targeting S. aureus with pathogen specific MAbs could promote wound healing in a polymicrobial setting, we developed a wound model in db/db^−/−^ type 2 diabetic mice with a mixture of S. aureus, P. aeruginosa and S. pyogenes. In a first attempt, intradermal (i.d.) inoculation with 1.0 × 10^5^ CFU (CFU) P. aeruginosa and S. pyogenes combined with 1.0 × 10^7^ SA resulted in large lesions (>400 mm^2^) 7 days postinoculation (Fig. S1 in the supplemental material), which were much larger than wounds resulting from the i.d. inoculation of one or two pathogens. These results indicated that polymicrobial infection was more severe than individual infections, but the inoculum of bacteria used was too rapidly progressive for a diabetic wound since mice succumbed from infection after 7 days (Fig. S1A). To reduce the disease severity of the three-pathogen mixture and replicate the chronic nature of a diabetic wound, the bacterial CFU were titrated down, and the model ultimately established with 1.0 × 10^7^ S. aureus, 1.0 × 10^5^ P. aeruginosa, and 1.0 × 10^1^ S. pyogenes. Although S. aureus/P. aeruginosa/S. pyogenes mixture- or S. aureus-injected mice exhibited similar skin lesion sizes (~100 mm^2^) 24 h postinoculation, S. aureus-induced lesions fully healed within 3 weeks while the mice injected with three bacteria mixture developed a wound that did not heal for up to 6 weeks (Fig. 1A). Additionally, inoculation with S. aureus/P. aeruginosa/S. pyogenes resulted in > 6 log outgrowth of S. pyogenes on day 7 relative to S. pyogenes injected alone (*P = *0.0007). While not statistically significant, a similar 4 log increase in P. aeruginosa was observed relative to P. aeruginosa alone (*P = *0.0711); however S. aureus CFU were similar in mice injected with S. aureus alone or S. aureus/P. aeruginosa/S. pyogenes (Fig. 1B). Nondiabetic lean mice (db/db^-/+^) inoculated with S. aureus/S. pyogenes/P. aeruginosa developed significantly smaller lesions compared to diabetic littermates (*P = *0.0002) (Fig. 2A and B). Bacterial growth in nondiabetic mice was also significantly reduced for S. aureus and S. pyogenes on day 14 (*P < *0.05, Fig. 2C), and the bacteria were no longer detectable after 21 days (Fig. 2C). These results suggest that in a diabetic host, a mixed skin infection potentiates bacterial growth and prevents healing compared to infection with individual pathogens.

10.1128/msphere.00130-22.1 FIG S1 Polymicrobial wound model with S. aureus (SA), S. pyogenes (SP) and P. aeruginosa (PA). Diabetic mice (*n* = 10) were i.d. inoculated with single organism, combination of two or three and lesion sizes measured over time. Bacteria numbers for inoculation were S. aureus (SA) SF8300 strain (1.0 × 10^7^ CFU), S. pyogenes (SP) (1.0 × 10^5^ CFU), P. aeruginosa (PA) (1.0 × 10^5^ CFU) (A) Inoculation with single organism SA, SP, PA or the SA+PA+SP/. (B) SA, SP or SA/SP, (C) SA, PA or SA/PA, (D) SP, PA or SP/PA. Download FIG S1, TIF file, 0.1 MB. Copyright © 2022 Tkaczyk et al. 2022 Tkaczyk et al. https://creativecommons.org/licenses/by/4.0/ This content is distributed under the terms of the Creative Commons Attribution 4.0 International license.

S. aureus AT has been reported to potentiate bacterial growth and increase disease severity in a mouse mixed bacterial lung infection model (40). AT is also reported to be a key virulence factor in S. aureus-skin and soft tissue infection models and could play a similar role in the diabetic polymicrobial wound model (23, 41, 42). Here, AT levels in skin lesions 24h post-injection of S. aureus alone were significantly higher in diabetic animals compared to lean littermates (Fig. 2D and *P* = 0.0097) and this difference was even more pronounced in the mixed polymicrobial wound (Fig. 2D *P = *0.0002, and Fig. S2 for multiple S. aureus clinical wound isolates). Collectively, these data demonstrate that AT expression is potentiated in diabetic mice and even more so in a polymicrobial wound, suggesting it may play a role in the delayed wound healing seen in this polymicrobial wound model, but the complex nature and multiple stages of a DFU may require targeting different aspects of S. aureus virulence.

10.1128/msphere.00130-22.2 FIG S2 AT production in vivo is potentiated in polymicrobial wounds. Mice (*n* = 10 per group) were i.d. inoculated with different S. aureus isolates (1.0 × 10^6^ CFU) alone or in combination with P. aeruginosa (1.0 × 10^5^ CFU) and S. pyogenes (10 CFU). Skin lesions were harvested from 5 mice per group after (A) 1 day or (B) 5 days, homogenized and AT quantified by ELISA from homogenates. AT levels represented in ng/mL/mg skin as mean ± standard deviation. Statistical differences for amount of AT produced one day after inoculation with S. aureus alone or with S. aureus/S. pyogenes/P. aeruginosa in diabetic mice was analyzed with an unpaired t test. (*P* values were 0.0078 *for SF8300, 0.0009** for 1422563, <0.0001*** for 1447526,1414516, and 1468003). *P* values were considered statistically different if <0.05. Download FIG S2, TIF file, 0.1 MB. Copyright © 2022 Tkaczyk et al. 2022 Tkaczyk et al. https://creativecommons.org/licenses/by/4.0/ This content is distributed under the terms of the Creative Commons Attribution 4.0 International license.

### Targeting S. aureus virulence factors with a specific MAb combination accelerates healing.

**FIG 3:**
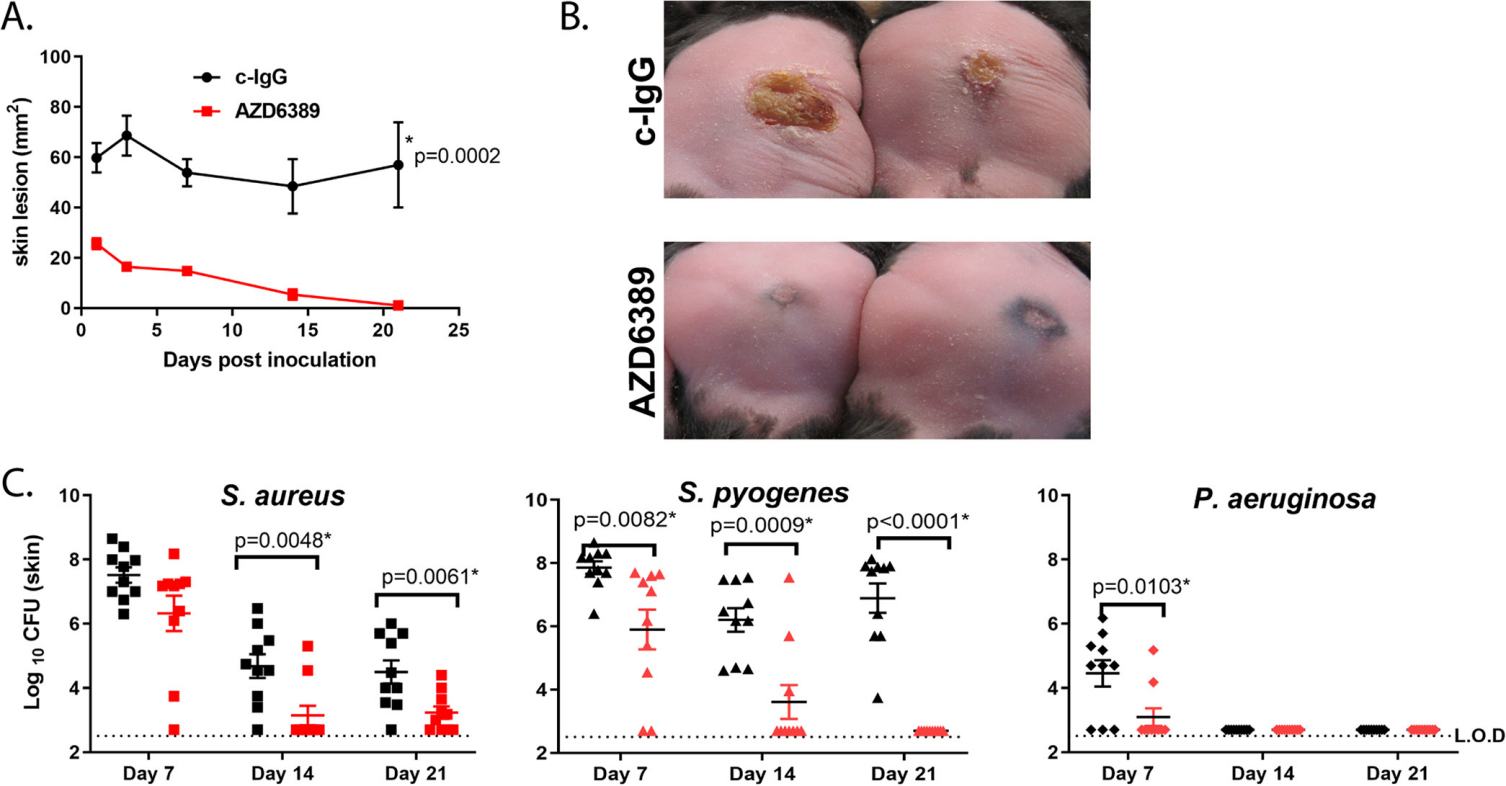
Targeting a single pathogen with AZD6389 accelerates polymicrobial wound closure. Diabetic mice (*n* = 10 per group) were passively immunized with AZD6389 (15 mg/kg each MAb) or c-IgG (15 mg/kg) and challenged 24 h later with S. aureus SF8300 (1.0 × 10^7^ CFU)/P. aeruginosa (1.0 × 10^5^ CFU)/S. pyogenes (10 CFU). (A) Skin lesion sizes were measured at indicated times and graphed as mean values ± SEM. Statistical difference between c-IgG and AZD6389 group was determined using a Vardi’s AUC test (two sample tests for growth under the curve dependent right censoring) and considered statistically different if *P < *0.05 as indicated with a (*). (B) Representative pictures of skin lesions 21 days after challenge. (C) Bacteria CFU in lesions at indicated times postchallenge. Data represent mean values ± standard deviation (error bars). Statistical difference for CFU between both group was analyzed with a Mann-Whitney test, and considered statistically different if *P < *0.05 as indicated with a (*).

DFU pathogenesis is complex and can represent different diseases starting with a superficial skin lesion progressing into deeper tissue and even into the bone which can lead to bloodstream infections and amputation (43). To target a single pathogen like S. aureus with a MAb-based approach, we hypothesized it would require multiple antibodies to neutralize different virulence factors to provide broad disease coverage and S. aureus isolate coverage in a complex disease such as DFU. We previously demonstrated that the administration of an α-AT and α-ClfA MAb combination was required to provide broad disease coverage in multiple S. aureus-induced preclinical models (25, 44). More recently, AZD6389 comprised of MEDI4893* (α-AT), AZD7745 (α-ClfA) and the anti-leukotoxin MAb AZD8887 (α-Leuk) demonstrated efficacy in mouse and rabbit surgical models (36, 37). We therefore tested whether targeting S. aureus with the multimechanistic MAb combination AZD6389 (described in Fig. S3 in the supplemental material) provided benefit in the diabetic polymicrobial wound model. Passive immunization with AZD6389 24 h prior to inoculation with a S. aureus/P. aeruginosa/S. pyogenes mixture resulted in full wound closure within 21 days, while wounds in mice that received a negative control IgG (c-IgG) remained unhealed out to 21 days (Fig. 1A and Fig. 3A). Moreover, neutralizing S. aureus virulence enabled the immune system to clear S. aureus and impaired S. aureus-mediated outgrowth of S. pyogenes and P. aeruginosa (Fig. 3C).

10.1128/msphere.00130-22.3 FIG S3 AZD6389 mechanism of action in vitro. (A) Neutralizing activity of human neutrophils intoxicated with LukSF (200 ng/mL), LukED (4000 ng/mL), HlgAB (2000 ng/mL) or HlgCB (200 ng/mL) in presence of serial dilutions of anti-Leuk MAb AZD8887 (dash line) or AZD6389 (plain line). No neutralization was observed against the 4 leukotoxins in presence of serial dilutions of negative control c-IgG (B) Neutralizing hemolytic activity of purified AT (10 ng/mL) on rabbit RBC in presence of serial dilutions of anti-AT MAb MEDI4893, AZD6389 or c-IgG. (C) Inhibition of fibrinogen binding to ClfA binding to fibrinogen in the presence of serial dilutions of anti-ClfA MAb AZD7745, AZD6389 or c-IgG. (D) Agglutination of S. aureus clinical isolates in the presence of human plasma. The graph illustrates the minimal concentration of AZD7745 or AZD6389 required to inhibit bacterial agglutination. Negative-control c-IgG did not show any inhibitory effect up to 150 μg/mL. (E) Bacterial OPK. S. aureus strain SF8300 was incubated with human HL-60 cells, human sera, and serial dilutions of AZD7745, AZD6389 or negative-control IgG. Download FIG S3, TIF file, 0.1 MB. Copyright © 2022 Tkaczyk et al. 2022 Tkaczyk et al. https://creativecommons.org/licenses/by/4.0/ This content is distributed under the terms of the Creative Commons Attribution 4.0 International license.

### All three MAb components of AZD6389 are required for wound closure in therapy.

**FIG 4:**
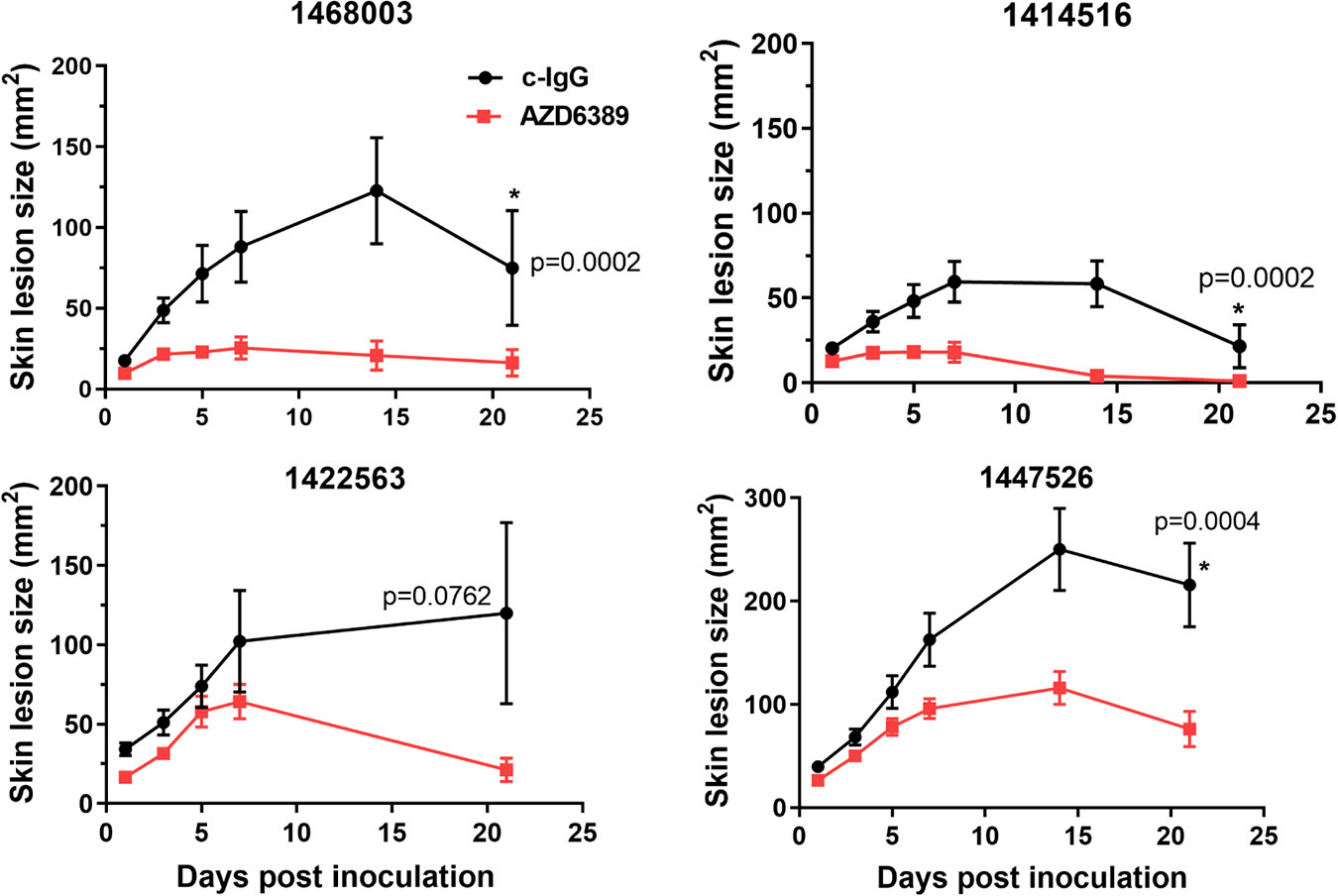
AZD6389 provides S. aureus strain coverage in the diabetic polymicrobial wound. Skin lesion size of mice (*n* = 10) inoculated with S. pyogenes (10 CFU)/P. aeruginosa (1.0 × 10^5^ CFU) and various S. aureus wound isolates (1.0 × 10^6^ CFU). AZD6389 (15 mg/kg each MAb) or c-IgG (15 mg/kg) was administered i.p 8 h later, and then 7 days later. Skin lesion sizes were measured at indicated days and represented as mean values ± standard errors mean (error bars). Statistical analysis between c-IgG and AZD6389 group was determined for each strain using a Vardi’s AUC test (two sample tests for growth under the curve dependent right censoring), and considered statistically different (*P < *0.05) as indicated with a (*).

**FIG 5:**
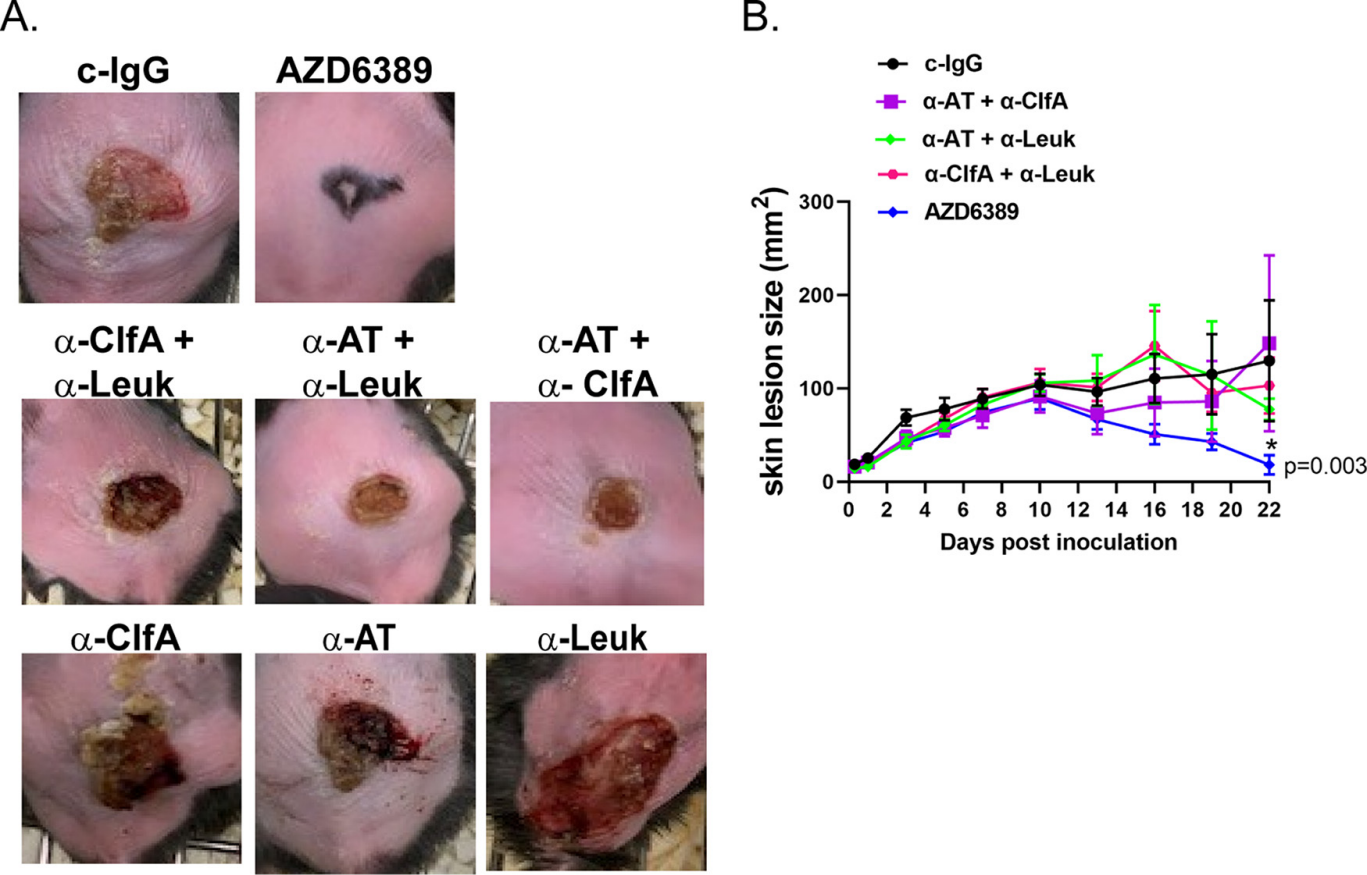
All three components of AZD6389 are required for closure of the polymicrobial wound. Diabetic mice (*n* = 10 per group) were inoculated with S. aureus 1447526 (1.0 × 10^6^ CFU)/P. aeruginosa (1.0 × 10^5^ CFU)/S. pyogenes (10 CFU), and i.p. immunized 8 h later with c-IgG, duo MAb combination or AZD6389 trio MAbs (all MAbs at 15 mg/kg). Animals were immunized 7 days later with same MAb combinations (A) Representative pictures of wounds 21 days postinoculation. (B) Kinetic of wound closure (lesion sizes) for c-IgG, duo combinations and AZD6389 treated groups. Lesion sizes were recorded at indicated times and represented as mean values ± standard errors (error bars). Statistical analysis between c-IgG and each group was determined using a Vardi’s AUC test (two sample tests for growth under the curve dependent right censoring), and considered statistically different (*P < *0.05) as indicated with a (*).

**FIG 6:**
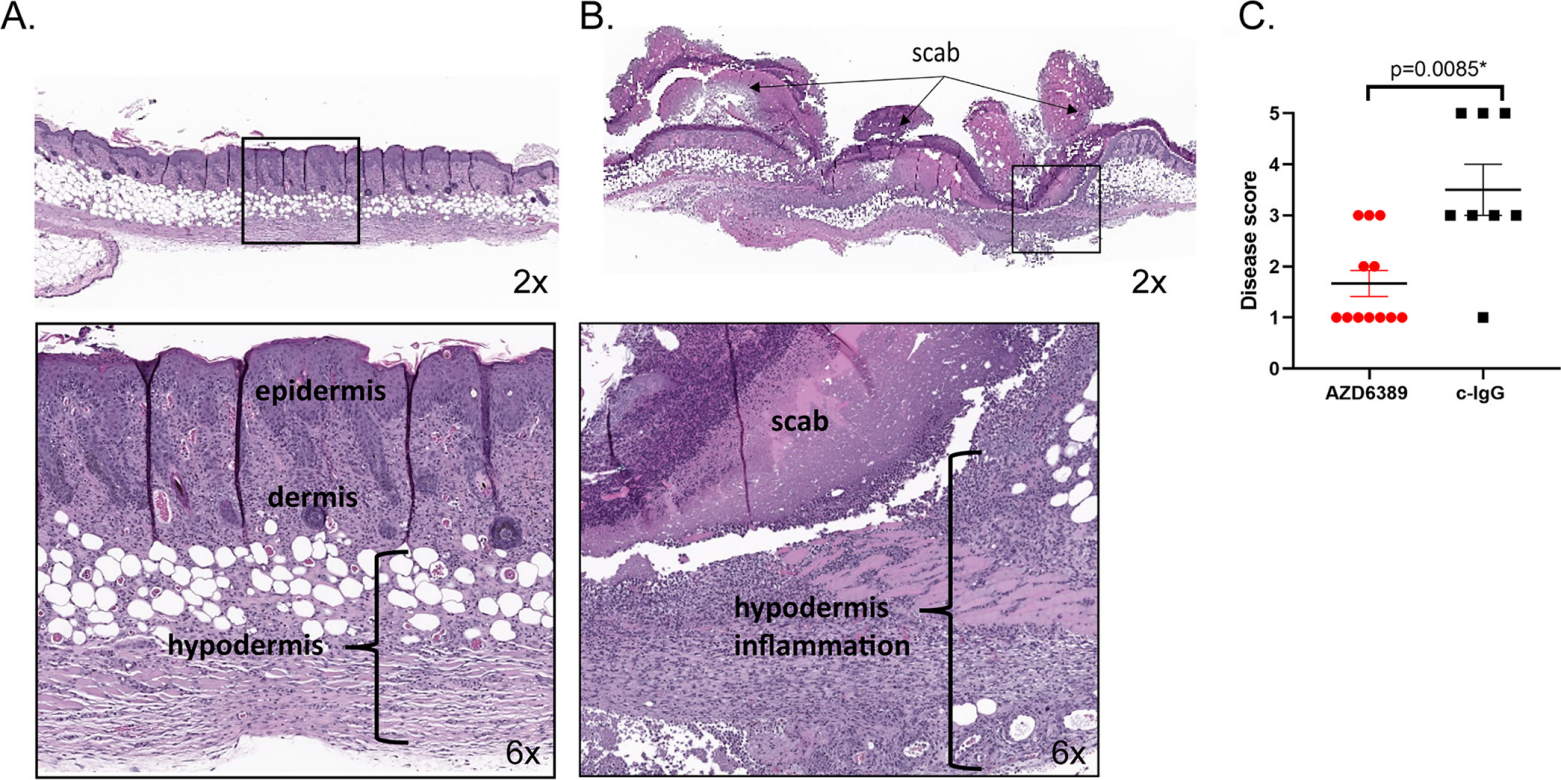
AZD6389 therapy provides full skin re-epithelization in the diabetic polymicrobial wound. Representative histology from mice (*n* = 12-8) inoculated i.d. with S. aureus 1447526 (1.0 × 10^6^ CFU)/P. aeruginosa (1.0 × 10^5^ CFU)/S. pyogenes (10 CFU). AZD6389 (15 mg/kg for each MAb) or c-IgG (15 mg/kg) were administered i.p. 8 h later, and then 7 days later. Each skin lesion was stained with H&E 21 days postinoculation. (A) Representative skin of AZD6389 treated mice showed epidermal closure (top left, 2X), with minimal inflammation and proliferation of fibroblast within the dermis and hypodermis (bottom left, 6X). (B) Representative skin of mice treated with c-IgG showed marked necrotic lesion under a serocellular scab (top right, magnification ×2) with full thickness inflammation, and necrotic cellular debris admixed with blood and bacterial colonies (bottom right, 6X). (C) Skin disease scoring (*n* = 12-8) ±SEM.

To determine if AZD6389 could provide a therapeutic benefit, the MAb combination was administered before or at different times after initiation of the polymicrobial wound. AZD6389 injection up to 8 h after inoculation accelerated closure of wounds initiated with different S. aureus wound isolates (Table S1 in the supplemental material) combined with S. pyogenes/P. aeruginosa in the polymicrobial wound model (Fig. S4 and Fig. 4). In this model, mice received 15 mg/kg of each anti-S. aureus MAb or 15 mg/kg of c-IgG. However, since no significant difference in kinetic of wound closure between c-IgG at 15 or 45 mg/kg groups were observed when administered in prophylaxis or 8 h postinoculation (Fig. S5A and Fig. S5B), c-IgG was used at 15 mg/kg for the study. To better understand the contribution and requirement of each MAb in wound healing, mice were treated with either the individual MAbs, a combination of two MAbs (α-AT+ α-ClfA, α-AT+ α-Leuk, or α-ClfA+ α-Leuk) or the three MAb combination AZD6389, 8 h post polymicrobial inoculation. Treatment with the combination of all three MAbs resulted in accelerated wound closure. In contrast, treatment with either the individual MAbs, the combination of two MAbs or c-IgG did not result in complete wound closure by day 21 (Fig. 5 and Fig. S6). These results demonstrate that all three MAbs are required to accelerate wound closure in diabetic mice. Histopathological examination of the skin after H&E staining demonstrated that AZD6389 treatment resulted in full closure after 21 days as shown by complete full-thickness re-epithelization and fewer inflammatory cells compared with skin from mice treated with c-IgG (Fig. 6 and Table S2). The effect of the anti-S. aureus MAb combination on bacterial CFU was quantified by measuring CFU 14 days after inoculation. AZD6389 significantly decreased S. aureus CFU (*P = *0.0042) as well as S. pyogenes (*P=* 0.0137) and P. aeruginosa (*P = *0.0054) compared to c-IgG (Fig. S7).

10.1128/msphere.00130-22.4 FIG S4 AZD6389 window of therapy. Diabetic mice (*n* = 10) were i.d. inoculated with S. aureus 1447526 strain (1.0 × 10^6^ CFU)/S. pyogenes (10 CFU)/P. aeruginosa (1.0 × 10^5^ CFU) and immunized i.p. at indicated time with AZD6389 MAb combination (each MAb at 15mg/kg) or c-IgG at 8 h post as negative control. As positive control, mice received AZD6389 (MAb at 15mpk each) 24 h before bacteria injection. Lesion sizes were measured up to 36 days post injection and graphed as mean values ± SEM. Statistical analysis between c-IgG and each group was determined using a Vardi’s AUC test (two sample tests for growth under the curve dependent right censoring). *P* values for c-IgG and AZD6389 (−24 h), (+4 h), (+8 h) were respectively 0.0004, 0.0012, 0.0016 and considered statistically different (*P < *0.05). *P* values for c-IgG vs AZD6389 (+24 h) was 0.6145. Download FIG S4, TIF file, 0.1 MB. Copyright © 2022 Tkaczyk et al. 2022 Tkaczyk et al. https://creativecommons.org/licenses/by/4.0/ This content is distributed under the terms of the Creative Commons Attribution 4.0 International license.

10.1128/msphere.00130-22.5 FIG S5 Comparison between c-IgG at 15 or 45 mg/kg for kinetic of wound closure. Diabetic mice (*n* = 10) were i.p immunized with AZD6389 MAb combination (each MAb at 15mg/kg) or c-IgG at 15 or 45 mg/kg (A) 24 h before or (B) 8 h after i.d inoculation with S. aureus 1447526 strain (1.0 × 10^6^ CFU)/S. pyogenes (10 CFU)/P. aeruginosa (1.0 × 10^5^ CFU). Mice received similar dose of antibodies 7 days after. Lesion sizes were measured up to 14 days post bacteria injection and graphed as mean values ± standard deviation. Statistical analysis between each group was determined using a Vardi’s AUC test (two sample tests for growth under the curve dependent right censoring), and indicated on the figure. Download FIG S5, TIF file, 0.1 MB. Copyright © 2022 Tkaczyk et al. 2022 Tkaczyk et al. https://creativecommons.org/licenses/by/4.0/ This content is distributed under the terms of the Creative Commons Attribution 4.0 International license.

10.1128/msphere.00130-22.6 FIG S6 All three components of AZD6389 are required for closure of the polymicrobial wound. Diabetic mice (*n* = 10) were inoculated i.d. with S. pyogenes (10 CFU)/P. aeruginosa (1.0 × 10^5^ CFU) and S. aureus 1447526 strain (1.0 × 10^6^ CFU), and i.p. immunized 8h later with c-IgG, anti-AT MAb, anti-Leuk MAb, anti-ClfA MAb or AZD6389 (all MAbs at 15mg/kg). Animals were immunized 7 days later with same MAb combinations. Lesion sizes were measured at indicated times up to 22 days post challenge and graphed as mean values ± standard deviation. Statistical analysis between c-IgG and each group was determined using a Vardi’s AUC test (two sample tests for growth under the curve dependent right censoring), and considered statistically different (*P < *0.05) as indicated with a (*). Download FIG S6, TIF file, 0.1 MB. Copyright © 2022 Tkaczyk et al. 2022 Tkaczyk et al. https://creativecommons.org/licenses/by/4.0/ This content is distributed under the terms of the Creative Commons Attribution 4.0 International license.

10.1128/msphere.00130-22.7 FIG S7 AZD6389 in therapy reduces bacteria outgrowth. Diabetic mice (*n* = 10) were id. inoculated with S. aureus 1447526 strain (1.0 × 10^6^ CFU)/S. pyogenes (10 CFU)/P. aeruginosa (1.0 × 10^5^ CFU) and immunized i.p. with AZD6389 or c-IgG (all MAbs at 15mg/kg) 8 h later and then 7 days after challenge. Skins was harvested 14 days pots inoculation and bacteria enumerated. Significant difference for CFU was analyzed with a Mann-Whitney test, and considered statistically different if *P < *0.05 as indicated with a (*). Graph is representative of four separate experiments. Download FIG S7, TIF file, 0.1 MB. Copyright © 2022 Tkaczyk et al. 2022 Tkaczyk et al. https://creativecommons.org/licenses/by/4.0/ This content is distributed under the terms of the Creative Commons Attribution 4.0 International license.

10.1128/msphere.00130-22.8 TABLE S1 Characteristics of the S. aureus clinical isolates. All strains were methicillin resistant (MRSA). Sequence type (ST), agr type, presence of genes alpha toxin (*hla*), bi-component leukotoxins HlgA, HlgB, HlgC (*hlgA, hlgB, hlgC*), LukS, LukF, LukD, LukE (*lukF, lukS, lukD, lukE*) was determined by analysis of whole genome sequencing data. Download Table S1, TIF file, 0.1 MB. Copyright © 2022 Tkaczyk et al. 2022 Tkaczyk et al. https://creativecommons.org/licenses/by/4.0/ This content is distributed under the terms of the Creative Commons Attribution 4.0 International license.

### AZD6389 reduced immune defects in diabetic wounds.

**FIG 7:**
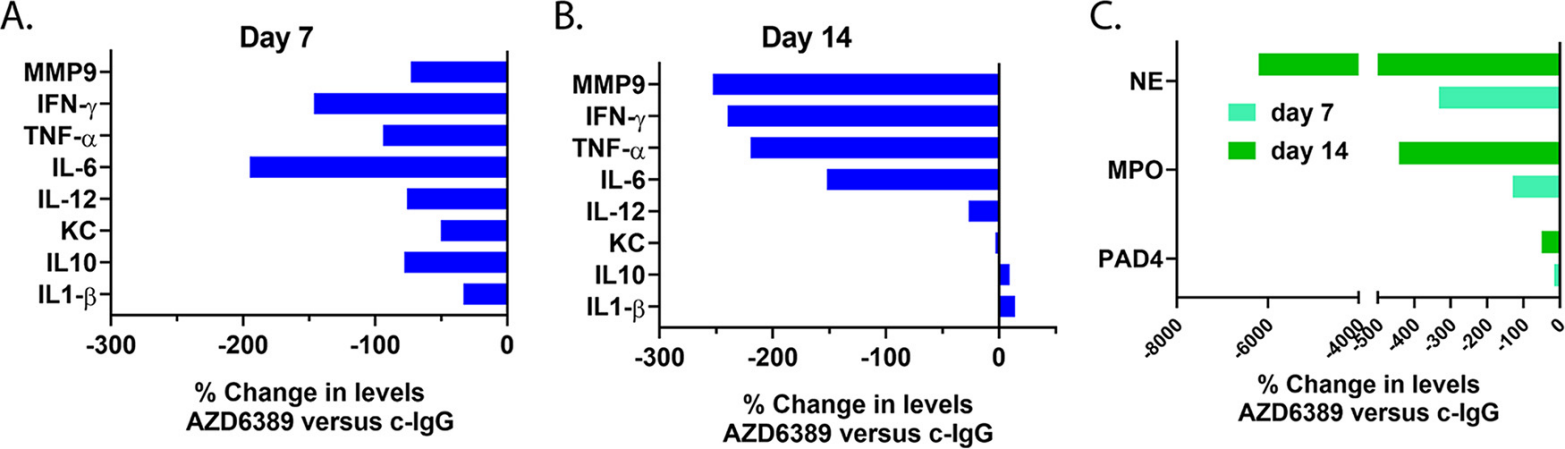
AZD6389 reduces the prolonged pro-inflammatory phase in the polymicrobial diabetic wound. Mice (*n* = 10) were inoculated i.d. with S. aureus 1447526 (1.0 × 10^6^ CFU)/P. aeruginosa (1.0 × 10^5^ CFU)/S. pyogenes (10 CFU). MAbs (15 mg/kg for each MAb) was administered i.p. 8 h later, and then 7 days later. Skin lesions were harvested at day 7 or 14 postinoculation, homogenized and homogenates analyzed for cytokines or metalloproteinase contents (A and B) or markers for NET formation (B). Data were normalized as % of change in levels for AZD6389 treated mice versus c-IgG.

Wound healing is a complex process involving coagulation, inflammation, cell proliferation and tissue remodeling (45). Diabetes is associated with immune defects including inflammatory dysregulation (46) and neutrophil activation (47, 48), which may delay the wound healing process. To determine the effect of AZD6389 on some aspects of the immune response, pro-inflammatory mediators were quantified in the skin lesions. Pro-inflammatory cytokines and matrix metalloproteinase 9 (MMP-9) were significantly decreased by the MAb combination compared to c-IgG 7 days postinoculation, with a more pronounced effect after 14 days for TNF-α and IFN-γ, suggesting that AZD6389 alleviated some of the prolonged pro-inflammatory response observed in the diabetic polymicrobial wound (Fig. 7A and B). Another hypothesis for delayed wound healing in diabetics is the propensity of neutrophils to form extra-cellular traps or NETs (49). Enzymatic activity of PAD_4_, a molecule controlling an intra-cellular pathway initiating NETosis along with neutrophil elastase (NE) and myeloperoxidase (MPO), two prototypical NET markers, were increased in the c-IgG treated mice over time and decreased in mice treated with AZD6389 (Fig. 7C). Together, these data demonstrated that neutralizing S. aureus virulence diminished the pro-inflammatory environment associated with nonhealing diabetic wounds.

## DISCUSSION

Microbial colonization, biofilm formation and infection are important factors linked to delayed healing of a diabetic foot ulcer (DFU) (12, 33, 50). DFU pathogenesis is complex and cover a broad range of diseases (43). Most DFUs are colonized with numerous bacteria (51), but S. aureus presence is associated with delayed healing and worse disease outcomes (52, 53). S. aureus possesses a large collection of virulence factors involved in skin and wound colonization, and can promote tissue damage, biofilm formation and disease progression (54). However, bacterial interactions and their role in nonhealing DFU are poorly understood. Here we demonstrate that i.d. inoculation of diabetic mice with three bacterial pathogens results in more severe wounds than inoculation with one or two pathogens and that targeting multiple S. aureus virulence factors (AT, the bicomponent leukotoxins, and ClfA) with AZD6389 accelerated wound healing in mice with polymicrobial wounds.

AT and the four bicomponent leukotoxins LukSF/LukED/HlgAB/HlgCB are pore forming toxins with a broad cell tropism that damage tissue and enable the bacteria to evade the host immune system (28, 55). Since P. aeruginosa has been reported to increase AT production in S. aureus/P. aeruginosa coinfected pig skin (56) and AT neutralization accelerates wound closure of S. aureus infected wounds in diabetic mice (57), we speculated that persistence in our polymicrobial diabetic wound was due in part to increased AT levels. We found that S. aureus expresses higher AT levels in diabetic mice than in nondiabetic littermates (Fig. 2), and even more in the presence of P. aeruginosa and S. pyogenes in the diabetic wounds (Fig. S2 in the supplemental material). We previously reported that S. aureus potentiated a mixed bacterial lung infection and promoted pathogen outgrowth in part through AT mediated impairment of alveolar macrophage phagocytic activity (40). AT may have a similar effect in the current model however, therapeutic administration of α-AT MAb MEDI4893 was not sufficient to promote wound healing, suggesting that other virulence factors are also involved in delayed healing in diabetic mice (Fig. 5 and Fig. S6).

Bacterial communities in chronic wounds form biofilms and downregulate genes involved in proliferation, resulting in reduced susceptibility to phagocytic killing and antibiotics (50). S. aureus biofilm formation may increase in a diabetic host since it exploits the hypercoagulable state in diabetics by upregulating surface expression of ClfA (58), a fibrinogen-binding adhesin essential for bacterial agglutination, biofilm and abscess formation as well as inhibition of complement-mediated phagocytosis (30). We recently demonstrated the complementary roles of AT and ClfA in the mouse hematogenous implant-related S. aureus biofilm infection model and determined that targeting both AT and ClfA (with MEDI4893 and AZD7745) provided benefit over the individual MAbs, highlighting the need for a MAb combination (36, 44). In a lethal mouse bacteremia model, we have demonstrated that the anti-AT/Anti-ClfA MAb combination required both the anti-ClfA human MAb opsonophagocytic killing activity and ability to prevent agglutination for protection (59). Wozniak et al. also showed that S. aureus biofilms released the LukSF and HlgAB leukotoxins to elicit NET formation and escape neutrophil-mediated phagocytosis (60). This may explain why only AZD6389, a multimechanistic MAb combination comprising an α-Leuk MAb combined with α-AT and α-ClfA MAbs which together inhibits bacterial agglutination, cell lysis and promotes opsonophagocytic killing (Fig. S3), was required for complete skin re-epithelization in a S. aureus/S. pyogenes/P. aeruginosa -inoculated diabetic wounds.

Functional defects of neutrophils (49) and macrophages (61) in diabetic individuals are associated with tissue damage and delayed wound healing. S. aureus secreted enzymes such as hyaluronidase (62) increases permeability of connective tissues and may amplify these defects by facilitating neutrophil and macrophage recruitment in the wound. Diabetes is associated with low grade inflammation which primes neutrophils for spontaneous NETosis (3, 46). It has been reported that AT stimulates NET formation by a low density neutrophil population enriched in diabetic animals (63). NETosis is also increased by TNF-α (46), a cytokine elevated and sustained in the polymicrobial wound (Fig. 7A and B). Therefore, by neutralizing AT and the leukotoxins and decreasing pro-inflammatory cytokines present in the polymicrobial wound micro-environment, AZD6389 reduced markers of NET formation as evidenced by decreases in PAD_4_, MPO, and NE (Fig. 7C).

Targeting S. aureus with a multimechanistic MAb combination provides several benefits. S. aureus expression of secreted toxins and ClfA dependent of the specific strain and their lifestyle and growth phase (27). By neutralizing multiple virulence factors AZD6389 could extend coverage throughout the different stages of DFU pathogenesis. In fact, the multimechanistic MAb combination and its component MAbs have been reported to provide protection in a variety of disease models that may represent different aspects of DFU pathogenesis as it progresses from a superficial wound into deeper tissues including the bone and in some case resulting in systemic bloodstream infections. These models include dermonecrosis, surgical wound infection, a bone implant infection and bacteremia in addition to the diabetic polymicrobial wound model reported here (23, 25, 36, 37, 44, 57). Together, the results reported herein support a role for S. aureus in the development of chronic polymicrobial diabetic wounds and specifically targeting S. aureus virulence with the multimechanistic MAb combination AZD6389 may open new therapeutic perspectives for all stages of DFU.

## MATERIALS AND METHODS

### Bacteria strains and growth.

Community-acquired methicillin-resistant S. aureus (CA-MRSA) USA300 SF8300 strain was previously described (59). Clinical S. aureus wound isolates 1414516, 1422563, 1447526, 1468003 were obtained from a collection of an international antibiotic resistance surveillance program. Basic demographic data (age, sex, hospital location, sample type) were provided for each isolate by International Health Management Associates (IHMA). Beta hemolytic S. pyogenes strain BAA-947 strain Rosenbach was purchased from ATCC (Manassas, VA). P. aeruginosa cytotoxic strain 6077 was provided by J. B. Goldberg (University of Virginia, Charlottesville, VA). Bacteria were grown to mid-log phase at an optical density at 600 nm (OD_600_) of 0.8 in Trypticase soy broth (TSB, VWR International), washed twice in ice cold PBS (Invitrogen), and frozen in 10% glycerol-TSB aliquots. S. aureus and S. pyogenes challenge inocula were prepared from one frozen vial for each experiment, diluted in ice-cold PBS pH 7.2 (VWR International), and placed on ice until injection. P. aeruginosa was streaked on a Tryptic soy agar (TSA) plate (VWR International), incubated overnight at 37°C and the challenge inoculum was prepared by diluting one single colony in PBS to 1 × 10^5^ CFU/mL.

### Whole-genome sequencing and genetic analysis.

DNA was purified from bacterial cultures via bead beating followed by extraction using a PureLink Genomic DNA minikit (ThermoFisher). Sequencing libraries were prepared by Covaris mechanical shearing followed by a NEBNext Ultra DNA library preparation kit for Illumina (New England BioLabs Inc.). Sequencing was performed via MiSeq 2 × 250 runs (Illumina) with a targeted depth of 150-fold. Multi-Locus Sequence Typing was performed by SRST2 (64) using a S. aureus MLST database downloaded from pubmlst.org on 08OCT2021. Read sets were screened for leukotoxins by direct‐read mapping implemented in SRST2 (64), using a 90% coverage cut‐off and reference genes obtained from USA300_FPR3757 GenBank: CP000255.1. Sequences were assembled *de novo* with SPAdes (65) and annotated with Prokka (66). Agr typing was performed by BLAST (67) of isolate agrC genes against representative agrC genes from each agr type: agr type I GenBank: AF210055; agr type II GenBank: AF001782; agr type III GenBank: AF001783; and agr type IV GenBank: AF288215 (68).

### Monoclonal antibodies and reagents.

All MAbs are human IgG1 isotype. Anti-AT (α-AT) MAb MEDI4893 (or LC10) and anti-clumping factor A (α-ClfA) MAb AZD7745 (or SAR114) were previously described (24, 25) Cross-neutralizing leukotoxin (anti-LukSF/LukED/HlgAB/HlgCB or α-Leuk) AZD8887 (SAN481) MAb was generated by Humabs (Bellinzona, Switzerland) through Antigen-specific Memory B cell Repertoire Analysis (AMBRA) technology as previously described (36). Anti-gp120 human IgG1 R347 MAb was used as negative control (23) and indicated as c-IgG.

### Mice.

All animal studies were approved by the AstraZeneca Institutional Animal Care and Use Committee, and they were conducted in an Association for Accreditation and Assessment Laboratory Animal Care (AAALAC)-accredited facility in compliance with U.S. regulations governing the housing and use of animals. Eight- to 9-week-old male diabetic mice (glucose level ≥500 mg/dL) of strain BKS.Cg-Dock7m Leprdb/+ +/J were purchased from Jackson Laboratories (Bar Harbor, Maine) developed spontaneously clinical sign with similar features than human type 2 diabetes. Age matched male nondiabetic C57BLKS/J animals were used as lean control. BKS.Cg-Dock7m Leprdb/+ -/J were used as lean controls.

### Polymicrobial wound Model.

Mice were shaved and treated with Nair on their back (VWR International**)** 48 h prior bacterial inoculation. Animals were intradermal (i.d.) injected with 50 μL of bacteria mixture prepared as described above and monitored daily for any sign of distress. Mice were immunized with MAbs intraperitoneal (i.p.), diluted in 500 μL of cold PBS, either prophylactically 24 h before inoculation therapeutically at different times post i.d. challenge.

Skin lesion sizes were recorded at indicated time points on each figure and lesion area calculated as described previously (23). Statistical analysis between groups were performed for corrected repeated measures. For each animal, area under the curve (AUC) was calculated, and comparison of AUC were performed between groups using a Vardi’s AUC test (Two-sample tests for growth curves under dependent right censoring) (69). Difference between groups for kinetic of skin lesion sizes were considered statistically different if *P < *0.05 and indicated with a (*).

All experiments were performed in accordance with institutional guidelines following experimental protocol review and approval by the Institutional Biosafety Committee (IBC) and the Institutional Animal Care and Use Committee (IACUC) at AstraZeneca.

### Bacteria enumeration.

Animals were euthanized at indicated times following bacteria ID challenge, and bacteria CFU were quantified from total skin lesions. Skins were placed in sterile tubes containing 1 mL cold PBS and processed with a homogenizer (Omni Prep multisample homogenizer; Omni International, Marietta, GA). Bacteria were then serially diluted and CFU for S. aureus, S. pyogenes and P. aeruginosa determined respectively after plating on TSA, Blood agar, and Orientation CHROMagar plates (Becton, Dickinson). The limit of detection for bacteria enumeration in our model was 200 CFU.

### Mediator quantification in skin lesions.

Skin lesions were harvested at the indicated times postinoculation and processed as in (23) for protein extraction. Pro-inflammatory cytokines were measured with an Mesoscale 9-Plex pro-inflammatory mouse cytokines kit (Mesoscale, Gaithersburg, MD). Matrix metallo-proteinase 9 (MMP9) was measured with an enzyme-linked immune-absorbent assay (ELISA) kit (R&D systems). Myeloperoxidase (MPO), neutrophil elastase (NE) and PAD_4_ were quantified with assay kits from Cayman Chemical (Ann Harbor, MI). Values were normalized to pg/mg skin and expressed as % change in levels for AZD6389-treated group versus c-IgG group.

### Histology.

Mice were euthanized at indicated time postinoculation and skin lesions harvested, fixed in buffered 10% formalin (VWR International) for 24 h, and paraffin embedded (Leica Microsystems). Four μm sections were stained with hematoxylin and eosin (H&E, Mercedes Medical) following standard histopathological techniques. Stained slides were digitally scanned on Aperio AT2 slide scanner (Leica BioSystems) at ×20 magnification and photomicrographs were taken at 2× and 6× magnifications. All stained sections were analyzed using a Nikon 90i brightfield microscope by a blinded board-certified pathologist and scored from 0 (*normal*) to 5 (*severe*).

### Quantification of alpha toxin in skin lesions.

Skin lesions were harvested with an 8-mm wound punch after 1 or 5 days of bacteria inoculation, and snap-frozen on liquid nitrogen. Skins were processed for protein extraction as detailed in (23). AT in lesions was quantified by ELISA. Maxisorp 96-well plates (Nunc) were coated with purified anti-AT MAb MEDI4893* (0.1 μg/mL) in 100 μL of 0.2M carbonate/bicarbonate buffer, and incubated overnight at 4°C. After three washes with PBS 0.1% Tween (wash buffer), plates were blocked at room temperature with 200 μL of PBS 2%BSA (Sigma). Following three washes, twofold serial dilutions in PBS (starting at 1:2) of digested skin supernatants were added to the plates under 50 μL and incubated for 90 min at room temperature with a 200 rpm orbital shaking. Purified AT was used as a standard (twofold serial dilutions starting at 200 ng/mL). After three washes, plates were incubated with polyclonal rabbit IgG anti-AT (2 μg/mL) in 50 μL PBS 0.1%BSA for 1 h at room temperature with a 200 rpm shaking. Following three washes, 50 μL of HRP-conjugated goat anti-rabbit IgG was added to the plates for 30 min at room temperature. After final washes, 100 μL of TMB substrate (KPL) was added, and the reaction was stopped after 8 min with 100 μL 0.2 M H_2_SO_4_. The optical density at 450 nm was measured with a spectrophotometer (Molecular Devices). AT quantity was expressed in ng/mL.

### Leukotoxin cytolytic assay on human neutrophils.

Human neutrophils were purified from heparin-drawn blood of three healthy anonymous volunteers (AstraZeneca employee blood donor program, male and female), as previously described (40). In a flat bottom white 96-well plate (Greiner), 25 μL of twofold serial dilution of anti-leukotoxin MAb AZD8887 or trio MAb AZD6389 were incubated for 15 min at room temperature with 25 μL of leukotoxin giving 90% of cell lysis, respectively of LukSF (200 ng/mL), LukED (4000 ng/mL), HlgAB (2000 ng/mL) or HlgCB (200 ng/mL).Plate was incubated for 2 h in a 37°C incubator with 5% CO_2_ after adding 50 μL of purified neutrophils to each well., followed by addition of 100 μL of Cell Titer Glo (Promega). After 30 min of shaking at 200 rpm, luminescent signal in relative luminescent unit (RLU) was measured with an Envision Multilabel plate reader (Perkin Elmer). Percentage of cell viability was calculated as follows: 100 − {100 * [(RLU_toxin + mAb_)/(RLU_cells alone_)]}.

### Alpha toxin (AT) hemolytic assay on rabbit red blood cells.

Rabbit red blood cell (RBC) hemolytic assay was performed as described previously (25). Briefly, serial dilutions of anti-AT MAb MEDI4893* or trio MAb AZD6389 (500 to 1.7 nM) were mixed with AT (0.1 μg/mL = 3 nM) in a U bottom 96-well plate (Thermo Fisher Scientific) and incubated with 50 μL of washed rabbit RBC (Peel Freeze) for 1 h at 37°C. Plates then were centrifuged at 1200 rpm for 3 min, and 50 μL of supernatant was transferred to new plates. Nonspecific human IgG1 R347 was used as a negative control (c-IgG). The optical density at 450 nm (OD_450_)was measured with a spectrophotometer (Molecular Devices). Percentage of inhibition of hemolysis was calculated as follow: 100 − [100* (OD_AT + MAb_/OD_AT, no MAb_)].

### Opsonophagocytic killing assay.

HL-60 cells were obtained from ATCC (Manassas, VA). HL-60 cells were cultured and differentiated as described (25). Cells were washed in saline and adjusted to 1.0 × 10^7^ cells/mL in high-glucose Hanks balance salt solution (HG-HBSS) (Invitrogen) with 0.1% gelatin (Sigma). Human serum collected from a healthy volunteer (AstraZeneca employee blood donor program) was adsorbed against S. aureus Reynolds capsule type 5 and S. aureus Wright capsule type 8 to deplete endogenous S. aureus-specific IgG and used as a complement source (1:100). USA300 SF8300 CA-MRSA clinical isolate was grown overnight in TSB, washed in cold saline, and diluted to 1e6 CFU/mL in saline. 10 μL of bacteria was incubated on ice for 30 min with 10 μL of anti-ClfA MAb AZD7745 or AZD6389 trio MAb serial dilution in 60 μL of HG-HBSS 0.1% gelatin. Ten microliters of sera and 10 μL of HL-60 were then added to the opsonized bacteria. Ten microliters of samples from each well were serially diluted in water with 0.1% saponin and dropped on a TSA plate (VWR International) before and after incubation for 1 h at 37°C with 100 rpm orbital shaking. Bacterial colonies were counted after 16 h of incubation of TSA plates at 37°C. The percentage of OPK was calculated as follows: 100 − [100* (CFU_at 1 h_)/(CFU_at time zero_)].

### Fibrinogen-ClfA binding assay.

Nunc MaxiSorp plates (Thermo Fisher Scientific) were coated overnight at 4°C with 2 μg/mL human fibrinogen (Sigma), washed with PBS containing 0.1% Tween 20 (wash buffer), and blocked for 1 h at room temperature (RT) with 200 μL/well casein (Thermo Fisher). Following three washes, the plates were incubated for 1 h at room temperature with a mix of 50 μL AviTag ClfA221–559 (2 μg/mL) and serial dilutions of anti-ClfA MAb AZD7745* or trio MAb AZD6389 in a 100 μL final volume of PBS. After three washes, bound ClfA was detected using horseradish peroxidase (HRP)-conjugated streptavidin (1:20,000; GE Healthcare) and then 100 μL of 3,3′,5,5′-tetramethylbenzidine (TMB) substrate (KPL). The reaction was stopped after 10 min with 100 μL 0.2 M H_2_SO_4_. The optical density at 450 nm (OD_450_) was measured with a spectrophotometer (Molecular Devices). The percent inhibition of ClfA binding to fibrinogen was calculated as follow: 100 − [100 * (OD_ClfA+MAb_/OD_ClfA,no MAb_)].

### Agglutination assay in human sera.

S. aureus clinical isolates were cultured overnight in 10 mL of TSB, washed in PBS, and suspended in 1 mL of ice-cold PBS. Anti-ClfA MAb AZD7745 or trio MAb AZD6389 were twofold serially diluted in 30 μL PBS starting at 200 μg/mL and mixed with 30 μL of citrated human plasma (AstraZeneca employee blood donor program) in a 96-well U-bottom plate (Thermo Fisher Scientific). Bacteria were added (30 μL) and incubated for 5 min at 37°C. Each well was evaluated visually, and the lowest MAb concentration where bacteria agglutinated was recorded. R347 was utilized as an isotype control human IgG1 (c-IgG).

### Statistics.

Statistical difference analysis was performed using Prism v9 (GraphPad). Data are represented as means standard errors of the mean (SEM), and values of *P < *0.05 were considered to be statistically significant.
