# Supplementary figures and images for: Neutralizing Staphylococcus aureus Virulence with AZD6389, a Three mAb Combination, Accelerates Closure of a Diabetic Polymicrobial Wound
Source: mSphere. 2022 Jun 1;7(3):e00130-22. doi: 10.1128/msphere.00130-22 (PMC9241520; doi:10.1128/msphere.00130-22)

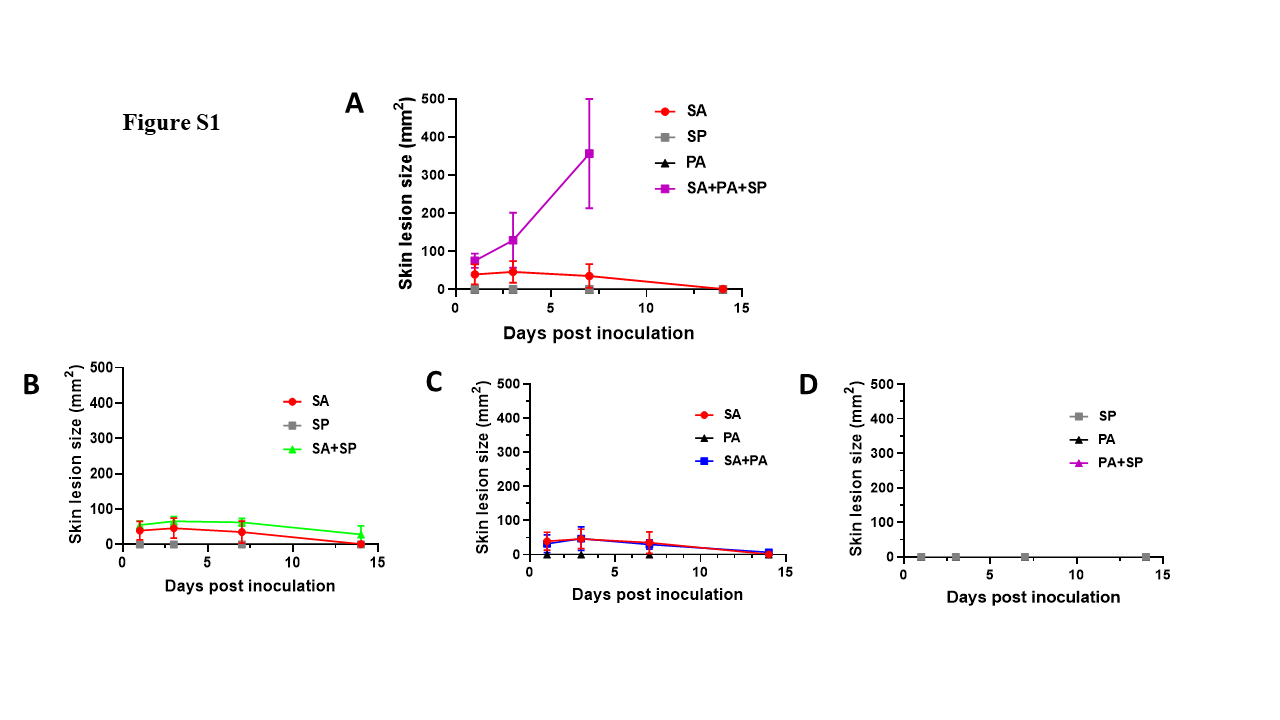

Supplement: FIG S1 [file msphere.00130-22-s0001.tif]

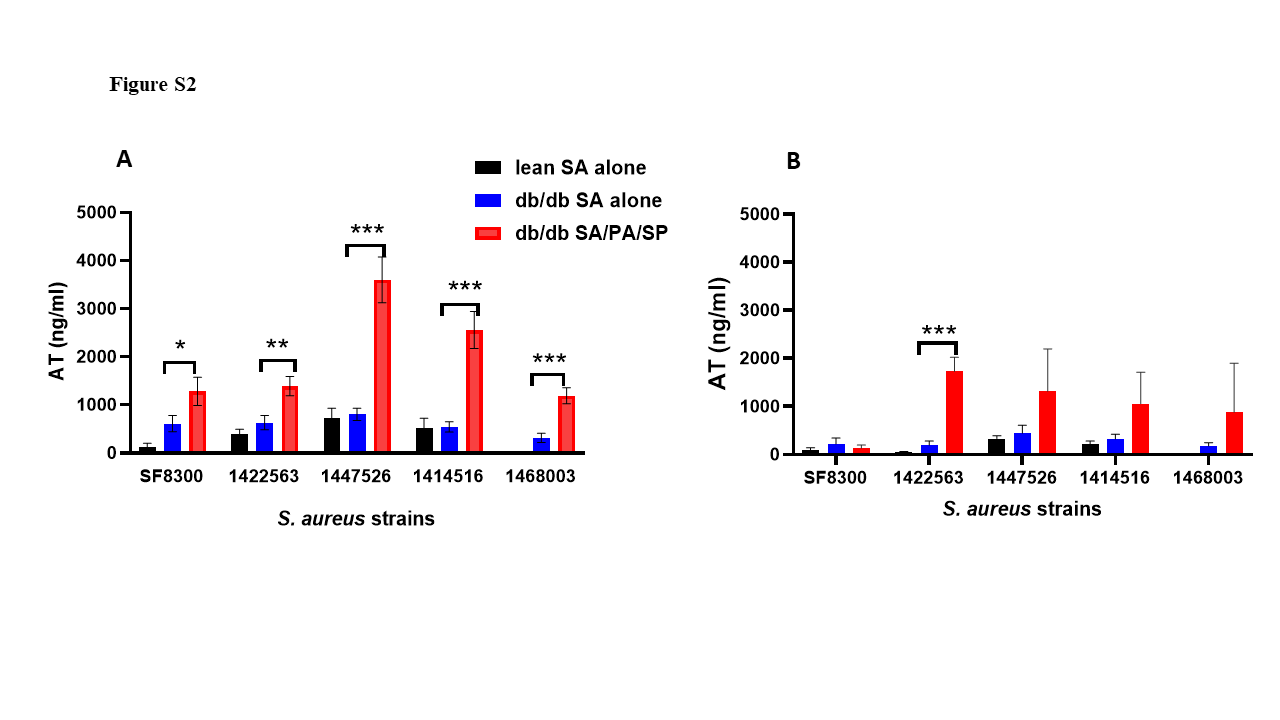

Supplement: FIG S2 [file msphere.00130-22-s0002.tif]

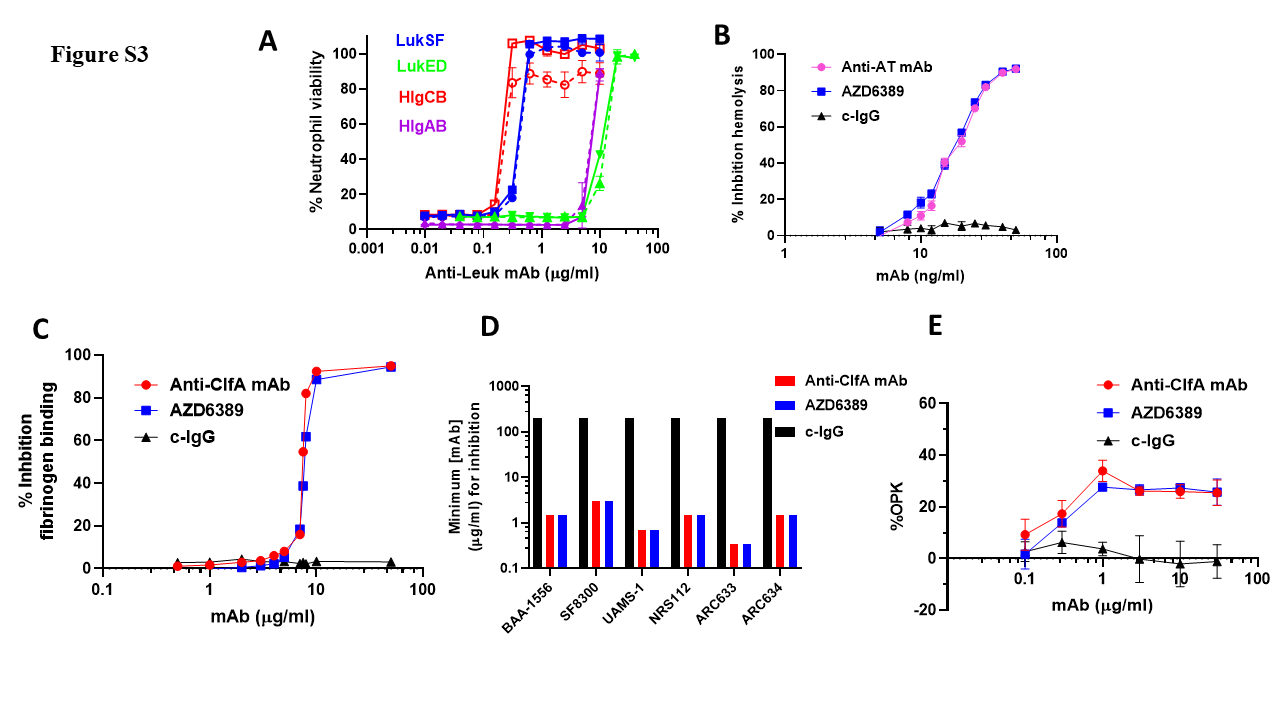

Supplement: FIG S3 [file msphere.00130-22-s0003.tif]

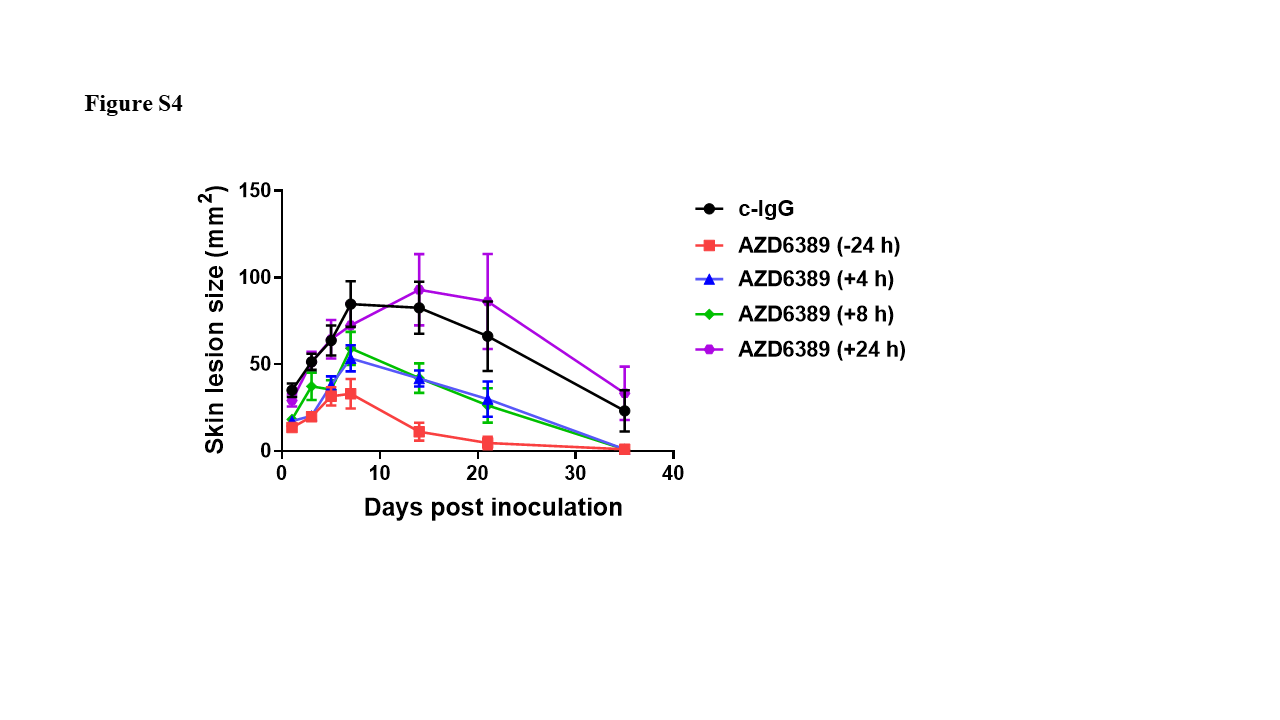

Supplement: FIG S4 [file msphere.00130-22-s0004.tif]

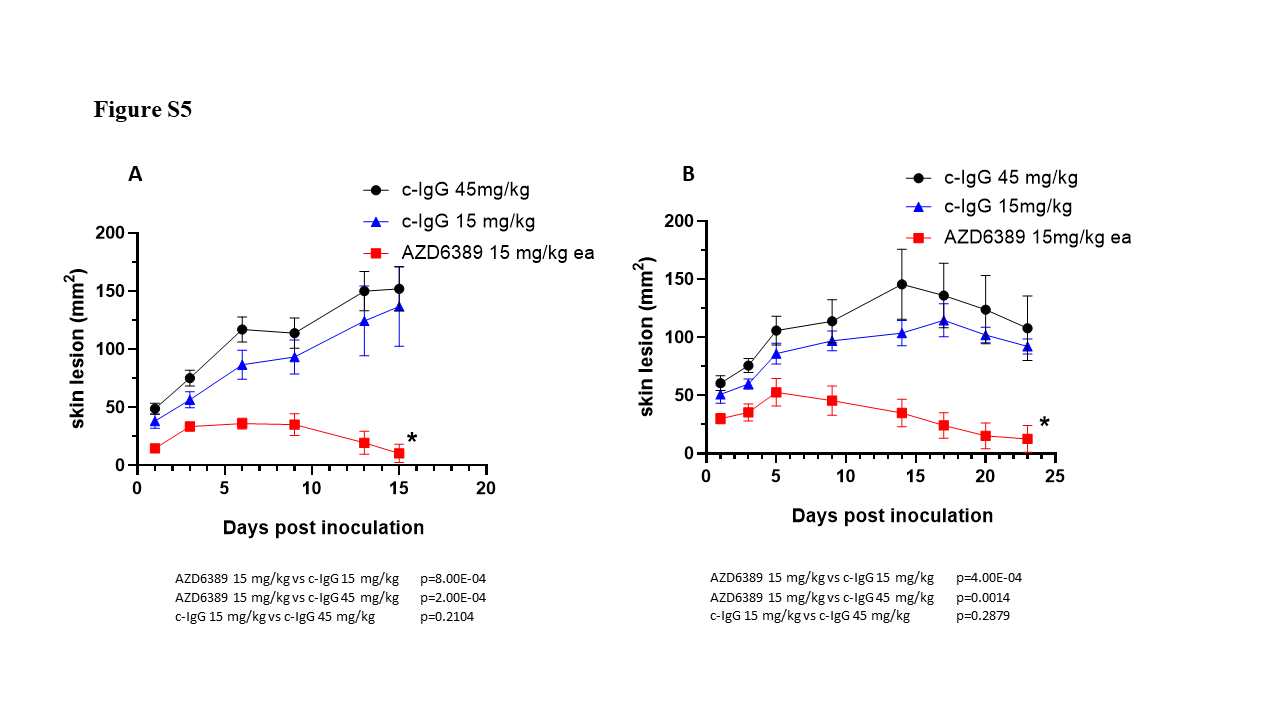

Supplement: FIG S5 [file msphere.00130-22-s0005.tif]

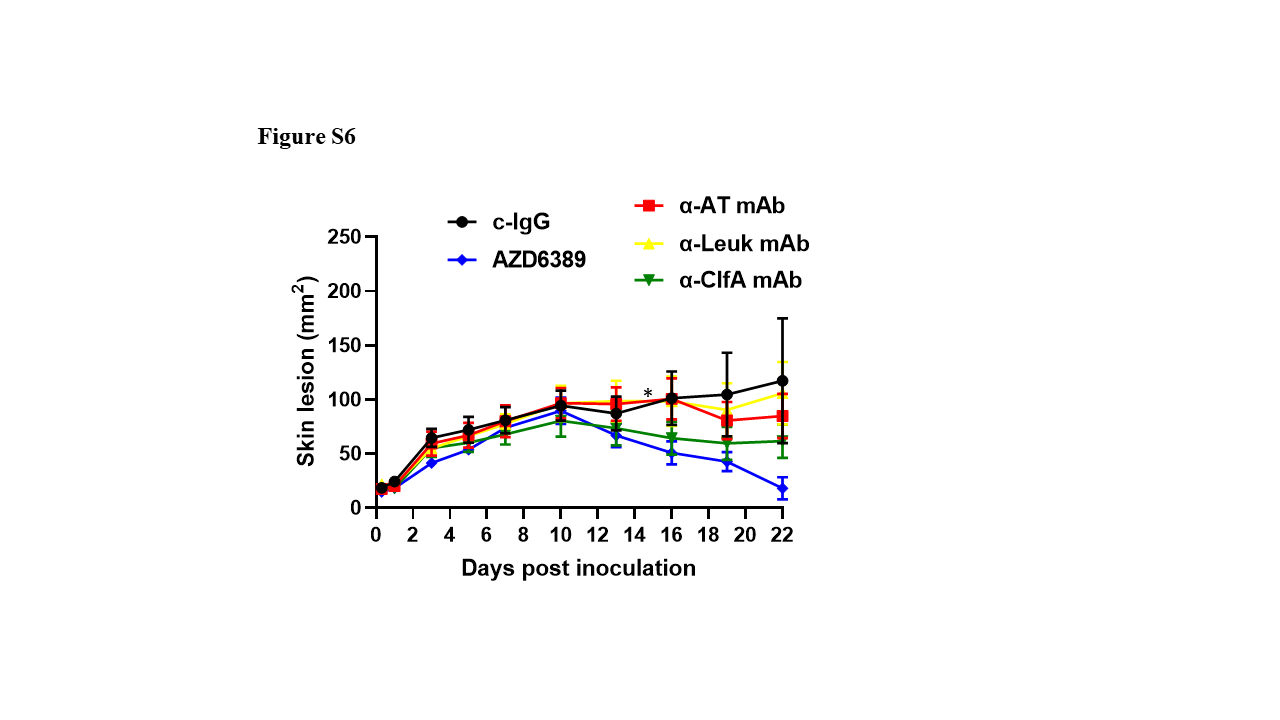

Supplement: FIG S6 [file msphere.00130-22-s0006.tif]

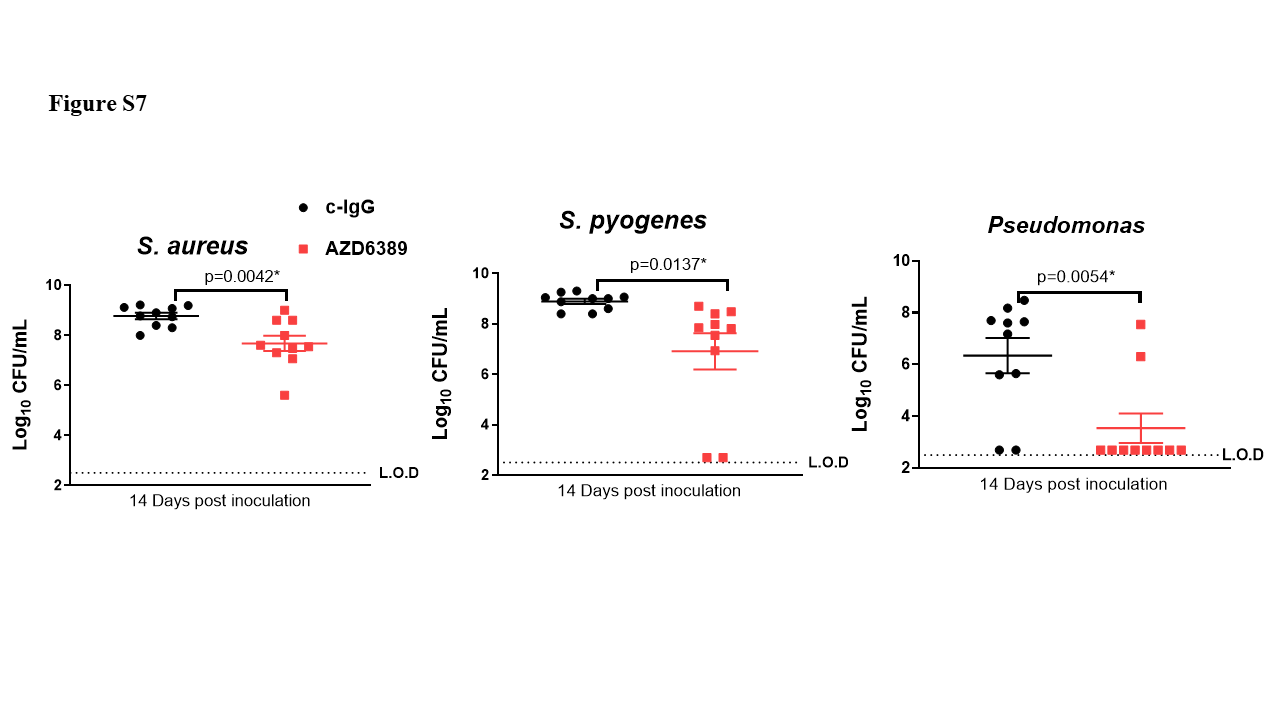

Supplement: FIG S7 [file msphere.00130-22-s0007.tif]

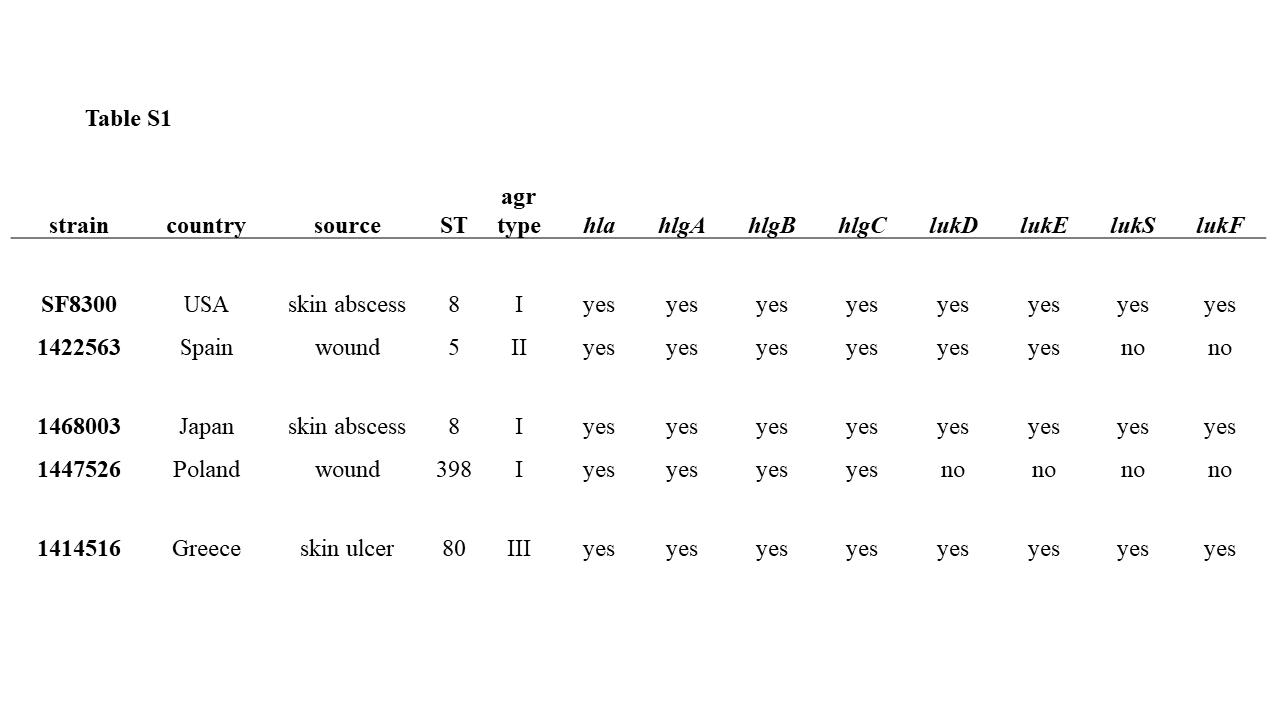

Supplement: TABLE S1 [file msphere.00130-22-s0008.tif]
